# Intravital lipid droplet labeling and imaging reveals the phenotypes and functions of individual macrophages in vivo

**DOI:** 10.1016/j.jlr.2022.100207

**Published:** 2022-04-06

**Authors:** Yue Li, Yuwei Du, Zhengqing Xu, Yuan He, Ran Yao, Huiran Jiang, Wen Ju, Jianlin Qiao, Kailin Xu, Tzu-Ming Liu, Lingyu Zeng

**Affiliations:** 1 School of Medical Technology, Xuzhou Medical University, Xuzhou, Jiangsu, China; 2 Blood Diseases Institute, Xuzhou Medical University, Xuzhou, Jiangsu, China; 3 Key Laboratory of Bone Marrow Stem Cell, Xuzhou, Jiangsu, China; 4 Department of Hematology, The Affiliated Hospital of Xuzhou Medical University, Xuzhou, Jiangsu, China; 5 School of Pharmacy, Xuzhou Medical University, Xuzhou, Jiangsu, China; 6 Faculty of Health Sciences, University of Macau, Taipa, Macao SAR, China

**Keywords:** macrophage, inflammation, lipid droplet, nanoparticle delivery, in vivo imaging, fatty acid analog, bone marrow, systemic inflammation, lipid trafficking, biomarker, APC, allophycocyanin, BM, bone marrow, DiD, DiIC_18_(5) solid (1,1'-dioctadecyl-3,3,3',3'-tetramethylindodicarbocyanine, 4-chlorobenzenesulfonate salt), IF, immunofluorescence, IL, interleukin, IVIS, In Vivo Imaging System, LD, lipid droplet, LPS, lipopolysaccharide, M-CSF, macrophage colony-stimulating factor, NOS, nitric oxide synthase, NP, nanoparticle, PE, phosphatidylethanolamine, PLGA, poly(lactic-co-glycolic acid), ROS, reactive oxygen species

## Abstract

Macrophages play pivotal roles in the maintenance of tissue homeostasis. However, the reactivation of macrophages toward proinflammatory states correlates with a plethora of inflammatory diseases, including atherosclerosis, obesity, neurodegeneration, and bone marrow (BM) failure syndromes. The lack of methods to reveal macrophage phenotype and function in vivo impedes the translational research of these diseases. Here, we found that proinflammatory macrophages accumulate intracellular lipid droplets (LDs) relative to resting or noninflammatory macrophages both in vitro and in vivo, indicating that LD accumulation serves as a structural biomarker for macrophage phenotyping. To realize the staining and imaging of macrophage LDs in vivo, we developed a fluorescent fatty acid analog-loaded poly(lactic-co-glycolic acid) nanoparticle to label macrophages in mice with high efficiency and specificity. Using these novel nanoparticles, we achieved in situ functional identification of single macrophages in BM, liver, lung, and adipose tissues under conditions of acute or chronic inflammation. Moreover, with this intravital imaging platform, we further realized in vivo phenotyping of individual macrophages in the calvarial BM of mice under systemic inflammation. In conclusion, we established an efficient in vivo LD labeling and imaging system for single macrophage phenotyping, which will aid in the development of diagnostics and therapeutic monitoring. Moreover, this method also provides new avenues for the study of lipid trafficking and dynamics in vivo.

Macrophages, a type of immune cells, almost reside in all tissues of body, from the skin to the bone marrow (BM) (1). Macrophages have remarkable plasticity, and they can be activated into specific subtypes by modifying their physiology and functions in response to local environmental cues. Activated macrophages are commonly divided into proinflammatory killing subtype and anti-inflammatory repairing subtype. Proinflammatory macrophages responding to bacteria, IFN-γ, and lipopolysaccharide (LPS) are involved in host defense and inflammation, whereas anti-inflammatory macrophages responding to interleukin-4 (IL-4), IL-10, and IL-13 play a pivotal role in tissue homeostasis and remodeling (2). Increasing evidence indicates that the reactivation of macrophages toward proinflammatory states under diverse kinds of stress is correlated with a plethora of inflammatory diseases, such as atherosclerosis, diabetes, obesity, rheumatoid arthritis, neurodegeneration, and BM failure syndromes (3, 4). Thus, characterization of macrophage activation status and the underlying molecular mechanism in situ will help elucidate their functions in these diseases; however, in vivo analysis of the macrophage activation status in their native multicellular microenvironment is challenging.

Although lipid droplets (LDs) have been initially described as intracellular fat storage organelles in adipocytes, increasing studies indicate that myeloid cells also form LDs under inflammation and stress (5, 6). Macrophages, as the effector cells of innate immunity, are found to form LDs to support their host defense when exposed to pathogens, such as parasites, bacteria, and viruses (7, 8, 9, 10, 11). However, abnormal LD accumulation in tissue-resident macrophages correlates with the pathogenesis of various inflammatory diseases. For instance, foam cells in atherosclerotic lesions can maintain the local inflammatory response by secreting proinflammatory cytokines (12, 13, 14). Moreover, LD-accumulating microglia contribute to neurodegeneration by producing high levels of reactive oxygen species (ROS) and secreting proinflammatory cytokines (15). These findings indicate that LD accumulation might be a hallmark of macrophages with proinflammatory functions.

In this study, based on the typical activation of in vitro BM-derived macrophages, we find that proinflammatory M_(LPS + IFN-γ)_ macrophages are characterized by LD accumulation, whereas resting macrophages and anti-inflammatory M_(IL-4)_ and M_(IL-10)_ macrophages do not contain any LDs. These features also hold for Matrigel plug-recruited macrophages and tissue-resident macrophages in mice. These findings demonstrate that LD accumulation could serve as a morphological index to distinguish proinflammatory macrophages from others.

It is feasible to distinguish LD-containing cells using imaging techniques, which has translational potential for identification of proinflammatory macrophages in vivo. However, current techniques for LD visualization are traditional in vitro staining method, and in vivo staining and imaging of LD in individual macrophages remains a challenge. Through nanocarrier screening, we selected the poly(lactic-co-glycolic acid) (PLGA) nanoparticles (NPs) as nanocarrier to deliver the lipophilic carbocyanine dye (DiI_C18_(5) solid (1,1'-dioctadecyl-3,3,3',3'-tetramethylindodicarbocyanine, 4-chlorobenzenesulfonate salt) [DiD]) and lipid staining dye (C1-BODIPY 500/510-C12) into macrophages. Using these dual fluorescence-labeled PLGA NPs, we achieved in situ and in vivo functional identification of single macrophages in various tissues under systemic or local inflammatory stress. Collectively, this study establishes an efficient in vivo labeling and imaging system of intracellular LDs for phenotyping the activation status and functions of individual macrophages in their dynamic niche, which is pivotal for disease diagnosis and preclinical research.

## Materials and methods

### Animals

C57BL/6 mice were obtained from the Beijing Vital River Laboratory Animal Technology Co Ltd. C57BL/6-Gt(ROSA)26Sor^tm1(CAG-tdTomato)^/Vst mice were obtained from the Beijing Vitalstar Biotechnology Co, Ltd. ApoE^−/−^ mice were kind gifts from Prof Jianlin Qiao (Xuzhou Medical University). The Animal Research Ethics Committee of the Xuzhou Medical University approved all experiments.

### Macrophage preparation and in vitro stimulation

Mouse BM cells from the tibia and femurs of mice were cultured in complete RPMI-1640 medium containing 10% denatured FBS, penicillin-streptomycin (100 U/ml), and 15% L929 cell-conditioned medium for 5 days to prepare BM derived-macrophages. On day 5, macrophages were harvested for further identification and experiments. For in vitro stimulation, macrophages were maintained in complete RPMI-1640 medium with 100 ng/ml LPS (catalog no.: L4391; Sigma-Aldrich) and 50 ng/ml IFN-γ (catalog no.: 575302; BioLegend), or 50 ng/ml IL-4 (catalog no.: 574304; BioLegend), or 50 ng/ml IL-10 (catalog no.: 575802; BioLegend), for 24 h.

### Flow cytometry

For in vitro-stimulated macrophages, macrophage maturation was assessed using allophycocyanin (APC) anti-mouse F4/80 (BM8; 1:80 dilution; BioLegend) and FITC anti-mouse/human CD11b antibodies (M1/70; 1:200 dilution; BioLegend). For in vivo BM macrophages, a single-cell suspension was prepared by flushing the BM cavity. Macrophages were identified using APC anti-mouse F4/80 antibody (BM8; 1:80 dilution; BioLegend) or FITC anti-mouse F4/80 antibody (BM8; 1:100 dilution; BioLegend) and V450 anti-mouse CD11b antibody (M1/70; 1:100 dilution; BD Biosciences). Macrophage activation status was assessed using phosphatidylethanolamine (PE) anti-mouse CD80 antibody (16-10A1; 1:100 dilution; eBioscience), PE/Cyanine7 anti-mouse CD206 antibody (C068C2; 1:80 dilution; BioLegend), and PE anti-mouse Nos2 (inducible nitric oxide synthase [NOS]) antibody (W16030C; 1:100 dilution; BioLegend). Data were acquired by flow cytometry on an LSRFortessa™ cell analyzer (BD Biosciences) and analyzed with FlowJo, version 10.1 (Tree Star).

### Macrophage function

Macrophages were stimulated as described above. Fluorescent probes dichlorodihydrofluorescein diacetate and diaminofluorescein-FM diacetate were used to measure the intracellular production of ROS and the activity of NOSs, respectively. Fluorescence intensity was detected using a Microplate Reader (Molecular Devices).

### Gene expression analysis

Total RNA was extracted from macrophages and purified using the TRIzol™ Reagent (catalog no.: 15596026; Invitrogen). Reverse transcription of RNA transcripts to complementary DNA was performed with the PrimeScript™ RT Reagent Kit (catalog no.: RR037A; TaKaRa). Quantitative real-time PCR assay was performed. Transcription levels were determined with 2× Universal SYBR Green Fast qPCR Mix (catalog no.: RK21203; ABclonal) using the LightCycler® 480 System (Roche). The expression levels of target genes were normalized to that of the housekeeping gene *β-actin*.

All primers were synthesized by GENEWIZ, and their sequences were as follows: *β-actin*, 5′-GTGACGTTGACATCCGTAAAGA-3′ (forward) and 5′-GCCGGACTCATCGTACTCC-3′ (reverse); *CD86*, 5′-TCAATGGGACTGCATATCTGCC-3′ (forward) and 5′-GCCAAAATACTACCAGCTCACT-3′ (reverse); *CD206*, 5′-CTCTGTTCAGCTATTGGACGC-3′ (forward) and 5′-TGGCACTCCCAAACATAATTTGA-3’ (reverse); *Arg1*, 5′-CATTGGCTTGCGAGACGTAGAC-3′ (forward) and 5′-GCTGAAGGTCTCTTCCATCACC-3’ (reverse); and *NOS2*, 5′-GAGACAGGGAAGTCTGAAGCAC-3′ (forward) and 5′-CCAGCAGTAGTTGCTCCTCTTC-3’ (reverse).

### Adoptive transfer and Matrigel plug assay

BM cells from C57BL/6-Gt(ROSA)26Sor^tm1(CAG-tdTomato)^/Vst mice were injected into the tail vein of irradiation pretreated C57BL/6 mice to generate BM chimeric mice (C57BL/6 mice with tdTomato BM). Two months after the adoptive transfer, Matrigel mixture was subcutaneously injected into the inguinal areas of mice. To induce activated macrophages in mice, 0.25 ml Matrigel (Corning) containing 100 ng/ml recombinant mouse macrophage colony-stimulating factor (M-CSF) (catalog no.: 576404; BioLegend) was used for naive macrophage differentiation and Matrigel containing M-CSF plus LPS + IFN-γ, IL-4, and IL-10 for in vivo activation.

For ex vivo imaging, Matrigel plugs were freshly dissected on the fifth day after plug implantation. Then, Matrigel plugs were incubated in the complete medium containing 1 μg/ml BODIPY 500/510 (catalog no.: D3823; Invitrogen) for 3 h. After incubation, Matrigel plugs were washed twice and mounted on a confocal dish for live-cell imaging. For immunofluorescence (IF) staining, Matrigel plugs were OCT embedded and sectioned for further examination.

### Preparation and characterization of dye-loaded NPs

Dye-loaded PLGA NPs were prepared using the antisolvent nanoprecipitation method (16). Briefly, PLGA (5 mg/ml) was dissolved in DMSO as organic phase. The BODIPY and DiD dye stock solution was added into the organic solution. Then, the organic phase was injected into the aqueous phase containing 0.1% Tween-80 in PBS (1:4, v/v). Under constant stirring at the speed of 400 rpm, the PLGA NPs loaded with fluorescence probes were prepared. After that, the organic solvent was removed using an ultrafiltration device with a centrifugation speed of 4,000 rpm for 10 min. The prepared PLGA NPs were diluted in Milli-Q water, and the particle size and zeta potential were measured using a Malvern Zetasizer Nano-ZS system (Malvern Instruments, UK). The morphology of PLGA NPs was examined by transmission electron microscopy (Tecnai G2 Spirit Twin).

For in vitro testing, macrophages were incubated in a complete medium containing dye-loaded PLGA NPs for 3 h and then 2 μg/ml Hoechst 33342 (MedChemExpress) for an additional 2 min. To evaluate the effect of NP uptake on LD accumulation, macrophages were incubated in a complete medium containing PBS-loaded PLGA NPs for 3 h and then sequentially stained with 1 μg/ml BODIPY 500/510 for 3 h and 2 μg/ml Hoechst 33342 for 2 min, respectively. Then, cells were washed twice, and the medium was replaced with complete medium for live-cell imaging.

### Confocal microscopy and live-cell imaging

Images were acquired using a Zeiss LSM 880 fluorescence confocal microscope (Zeiss, Germany) with fixed lasers (405, 488, 561, and 633 nm). For in vitro macrophage imaging, macrophages were incubated in the complete medium containing 1 μg/ml BODIPY 500/510 for 3 h and then 2 μg/ml Hoechst 33342 for an additional 2 min. After incubation, cells were washed twice and replaced with complete medium for live-cell imaging. We acquired BODIPY or FITC images at 488 nm excitation, Hoechst 33342 or V450 images at 405 nm excitation, and APC or DiD at 633 nm excitation. Images were subsequently imported into ImageJ software (NIH) for analysis.

### Immunocytochemistry staining

To examine the effect of LPS challenge on the LD formation of BM macrophages, single cells from the BM cavity were incubated in complete medium containing 1 μg/ml BODIPY 500/510 for 3 h and then with V450 anti-mouse CD11b antibody (M1/70, 1:100 dilution; BD Biosciences) and APC anti-mouse F4/80 antibody (BM8; 1:80 dilution; BioLegend) for an additional 30 min. After incubation, cells were washed twice, and the medium was replaced with complete medium for live-cell imaging.

### IF staining

Organs (bone, liver, lung, and adipose tissue) were freshly dissected and fixed in 4% paraformaldehyde for 6 h at 4°C. Then, the organs were washed with PBS overnight at 4°C with shaking to remove paraformaldehyde. After dehydration with 30% sucrose overnight, the organs were embedded in OCT and bisected by a Leica cryostat (Leica, Germany). Tissue sections were washed with PBS to remove OCT and blocked with 2% BSA for 30 min. Anti-mouse F4/80 antibodies (Cl:A3-1; Bio-Rad) were incubated overnight at 4°C, followed by the secondary antibody for 30 min at room temperature, and Hoechst 33342 for 30 min at room temperature. After incubation, tissue sections were washed twice with PBS for imaging.

### Intravital imaging

To observe the status of LD accumulation in tissue-resident macrophages under acute inflammation, 0.8 mg/kg LPS (O55:B5; Sigma-Aldrich) was used to induce systemic inflammatory stress via intraperitoneal injection. To observe the status of LD accumulation in tissue-resident macrophages under chronic inflammation, ApoE^−/−^ mice were fed with high-fat diet (containing 20% fat and 1.25% cholesterol) for 12 weeks to prime local adipose tissue inflammation. DiD/BODIPY-loaded PLGA NPs (in 200 μl PBS) were intravenously injected to label macrophages in vivo. On the second day post-NP injection, confocal microscopy was performed for ex vivo imaging and intravital imaging.

For ex vivo imaging, C57BL/6 mouse tissues (tibia marrow, liver, and lung) and ApoE^−/−^ mouse tissues (adipose tissues) were freshly dissected and incubated in the complete medium containing 2 μg/ml Hoechst 33342 for 30 min. After incubation, organs were washed twice and mounted on a confocal dish with Matrigel for live-cell imaging.

*In vivo* calvarial BM imaging was performed as described previously, with minor modifications (17). C57BL/6 mice were anesthetized by intraperitoneal injection of ketamine (150 mg/kg) and xylazine (10 mg/kg) for intravital imaging. A circular area of skin on the skull was excised to expose the underlying dorsal calvarial surface. Mice were subsequently immobilized on a custom-designed holder with a glass coverslip bottom. The exposed dorsal surface of the skull was superfused with sterile PBS and mounted on a coverslip for intravital imaging. We acquired BODIPY images at 488 nm excitation, Hoechst images at 405 nm excitation, and DiD images at 633 nm excitation. Images were subsequently imported into ImageJ software for analysis.

### In vivo imaging system

DiD-loaded PLGA NPs (in 200 μl PBS) were intravenously injected to label macrophages in vivo. On the second day post-NP-injection, the major organs were freshly dissected to evaluate the biodistribution of NPs. Fluorescence imaging was performed using the In Vivo Imaging System (IVIS®) Lumina S5™ (PerkinElmer).

### Statistical analysis

Data were evaluated with GraphPad Prism software (version 6.0; GraphPad Software, Inc) using one-way ANOVA with multiple comparison tests. Data are presented as mean ± SD. Differences were considered significant when *P* values were below 0.05.

## Results

### Proinflammatory macrophages are characterized by LD accumulation in vitro and in situ

**Fig. 1:**
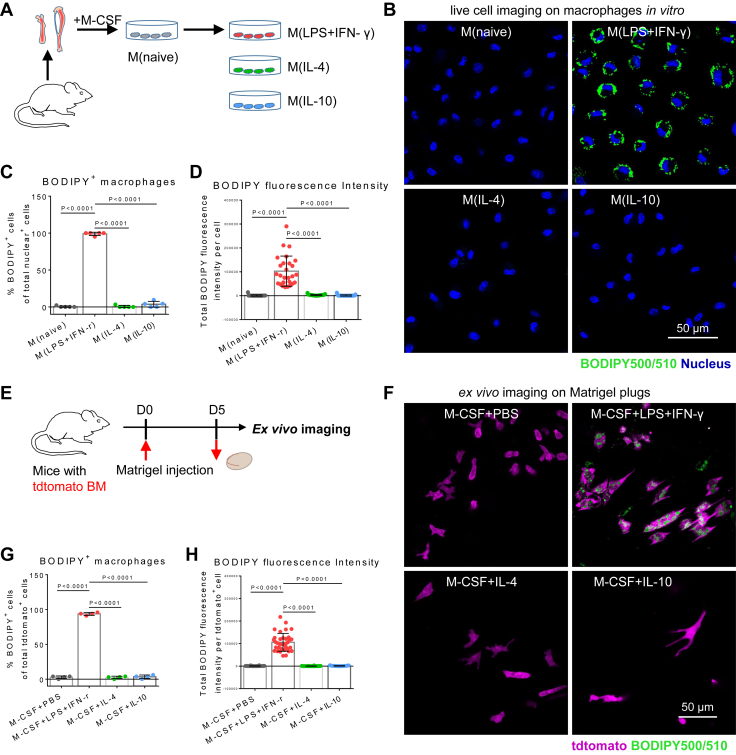
Proinflammatory macrophages feature LD accumulation in vitro and in vivo. A: Schematic diagram showing the culture and activation of BMDMs. B: Representative fluorescence confocal microscopy images of LDs (green color, BODIPY) and nuclei (blue color, Hoechst 33342) in macrophages. The scale bar represents 50 μm. C: Quantification of BODIPY+ (LD-containing) macrophages in all cells. D: ImageJ software was used to analyze BODIPY fluorescence intensity of individual macrophages. E: Schematic overview of experiment procedures. Matrigel mixture was injected into the inguinal areas of mice with tdtomato bone marrow cells. On day 5 after implantation, Matrigel plugs were harvested for ex vivo imaging. F: Representative fluorescence images of LDs (green color, BODIPY) in macrophages (purple color, tdtomato) recruited by Matrigel plugs. The scale bar represents 50 μm. G: Quantification of BODIPY+ (LD-containing) macrophages in tdtomato-positive cells. H: ImageJ software was used to analyze BODIPY fluorescence intensity of individual macrophages in Matrigel plugs.

Although many studies have highlighted the role of LD formation in the functional reprogramming of immune cells (18, 19, 20), LD biogenesis in differentially activated macrophages has not been studied functionally. To identify the differences of LD formation in activated macrophages, mouse BM cells were used to develop primary naive macrophages (illustrated in Figs. 1A and S1A) (21). Then, M_(naive)_ macrophages were converted into proinflammatory M_(LPS + IFN-γ)_ macrophages and anti-inflammatory M_(IL-4)_ and M_(IL-10)_ macrophages by exposure to different stimuli (Fig. 1A). Phenotyping based on gene expression (supplemental Fig. S1B), ROS production (supplemental Fig. S1C), and NOS activity (supplemental Fig. S1D) showed that M_(naive)_ macrophages were polarized to achieve the expected activated status.

C1-BODIPY 500/510-C12 is a fluorescent fatty acid analog used for staining LDs of live cells, live tissues, and live *Caenorhabditis elegans* via the metabolic incorporation route (22, 23, 24, 25, 26, 27). In this work, BODIPY dye was used for LD staining in activated macrophages. Using confocal microscopy, we found that proinflammatory M_(LPS + IFN-γ)_ macrophages exhibited high accumulation of LDs (Fig. 1B). This is consistent with previous findings that multiple cues, such as IFN-γ or LPS, can induce LD formation in macrophages (7, 18, 19). However, resting M_(naive)_ macrophages and noninflammatory M_(IL-4)_ or M_(IL-10)_ macrophages did not contain any LDs (Fig. 1B). Quantitatively, about 99% of M_(LPS + IFN-γ)_ macrophages were positive for BODIPY staining relative to M_(IL-4)_ and M_(IL-10)_ macrophages (Fig. 1C, D).

Considering the differences between in vitro 2D culture and in vivo 3D microenvironment, it is necessary to investigate the features of LD accumulation between activated macrophages are still significant in vivo. We used Matrigel mixtures containing M-CSF to attract circulating blood monocytes and to contribute their differentiation into macrophages in vivo. Meanwhile, the designed stimulating cytokines in Matrigel plugs educated macrophages in mice. On the fifth day after subcutaneous implantation, Matrigel plugs were harvested and stained for analysis (illustrated in Fig. 1E). The results of IF staining revealed that the cells in Matrigel plugs were F4/80-positive macrophages (supplemental Fig. S1E). To characterize the LD accumulation in these macrophages, we stained Matrigel plugs with free BODIPY. Using confocal microscopy, we found that M_(LPS + IFN-γ)_ macrophages still exhibited high accumulation of LDs, and M_(IL-4)_ and M_(IL-10)_ macrophages did not contain any LDs (Fig. 1F–H). The morphological features of proinflammatory macrophages are still separable from noninflammatory macrophages. These findings suggest that proinflammatory macrophages are characterized by LD accumulation in vitro and in vivo, and that this structural marker is feasible for distinguishing between them using imaging techniques.

### NP screening for in vivo staining of LDs in macrophages

Accordingly, the visualization of LDs will enable identification of macrophage activation status and elucidation of its role in different contexts. There are many imaging tools for LD visualization, including label-free imaging and fluorescence imaging (28). However, the subcellular visualization of LD dynamics in targeted single cells in vivo is still difficult to achieve. To label macrophage LDs in vivo, free BODIPY was injected via the intravenous route (supplemental Fig. S2A). Using flow cytometry, we found that the BODIPY fluorescence intensity of F4/80+ CD11b+ BM macrophages was not elevated after BODIPY injection relative to control mice, indicating that free BODIPY injection could not label the targeted macrophages and their LDs in vivo (supplemental Fig. S2B). It is thus clear that a nanocarrier is required to deliver the BODIPY dye into macrophages selectively in vivo.

Inspired by the phagocytosis function of macrophages, we sought to develop a novel dye-loaded NP to label the LDs of macrophages selectively in vivo. Liposomes are the most widely investigated delivery system for macrophage-targeted therapies owing to their low immunogenicity and high biocompatibility (29). Thus, we first loaded BODIPY into liposomes for LD staining. Surprisingly, we found that liposomes induced LD formation in resting or M_(IL-4)_ macrophages (supplemental Fig. S2C), indicating that liposomes could not be used for the delivery of lipid-staining dyes.

**Fig. 2:**
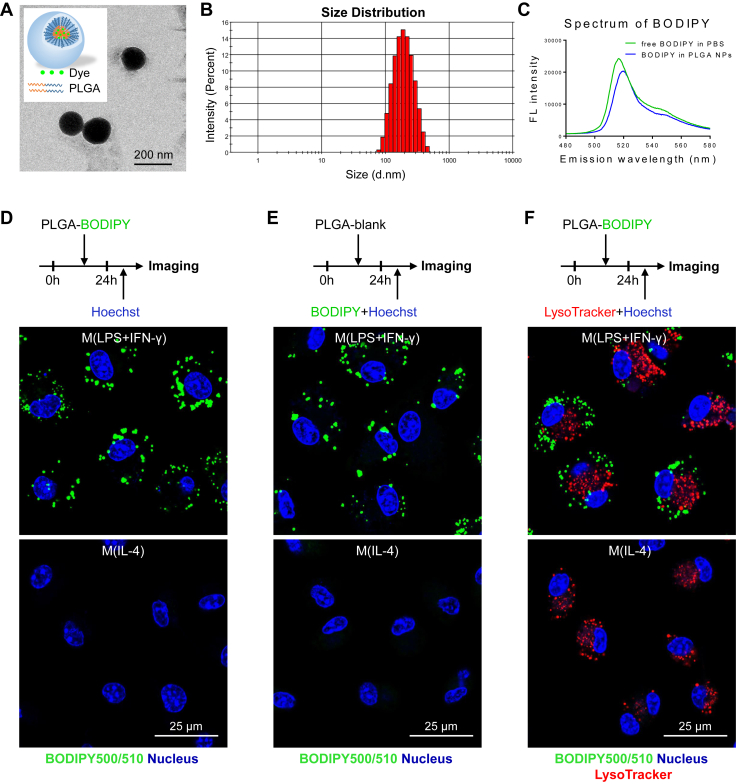
Characterization of BODIPY-loaded PLGA NPs in vitro. A: Schematic structure of dye-loaded PLGA NPs. Representative transmission electron microscopy images of BODIPY-loaded PLGA NPs; the scale bar represents 200 nm. B: The size distribution of BODIPY-loaded PLGA NPs. C: The fluorescence spectra of BODIPY dyes in PBS solution (green curve) and in the BODIPY-loaded PLGA NPs (blue curve). D: The labeling efficiency of BODIPY-loaded PLGA NPs in vitro. Fluorescence confocal images of labeled LDs in macrophages; LDs (green color, BODIPY) and nuclei (blue color, Hoechst 33342); the scale bar represents 25 μm. E: To examine the effect of PLGA NP engulfment on intracellular LD formation, blank PLGA NPs were administered into in vitro cultured macrophages; then, the cells were counterstained with free BODIPY. LDs (green color, BODIPY) and nuclei (blue color, Hoechst 33342); the scale bar represents 25 μm. F: Fluorescence confocal images of labeled LDs and lysosomes in macrophages; BODIPY-loaded PLGA NPs were administered into in vitro cultured macrophages; then, the cells were counterstained with LysoTracker Red and Hoechst 33342. LDs (green color, BODIPY), lysosomes (red color, LysoTracker), and nuclei (blue color, Hoechst 33342); the scale bar represents 25 μm.

Next, we tested another widely used NP for macrophage targeting, PLGA NPs (30). PLGA nanotechnology has been approved by the US Food and Drug Administration for drug delivery, diagnostics, and other applications in clinical and basic science research (31). BODIPY-loaded PLGA NPs were prepared as described before (Fig. 2A) (16). The mean diameter and mean zeta potential of the PLGA NPs was around 178 nm (Fig. 2A, B) and −41.6 mV when dissolved in water (supplemental Fig. S2C). The results of fluorescence spectra indicated that BODIPY was successfully loaded in these NPs (Fig. 2C). After engulfment by cells, the BODIPY-loaded PLGA NPs successfully labeled the LDs in activated macrophages (Fig. 2D). Moreover, PBS-loaded PLGA NPs did not cause obvious alterations in LD formation in activated macrophages (Fig. 2E). Using confocal microscopy, we found that BODIPY-labeled LDs and LysoTracker-labeled lysosomes did not colocate, indicating that the BODIPY dye was successfully intracellularly released from intact NPs after endocytosis (Fig. 2F). Collectively, PLGA NPs are a preferable delivery system for in vivo macrophage LD labeling.

### PLGA NPs selectively target macrophages in vivo

**Fig. 3:**
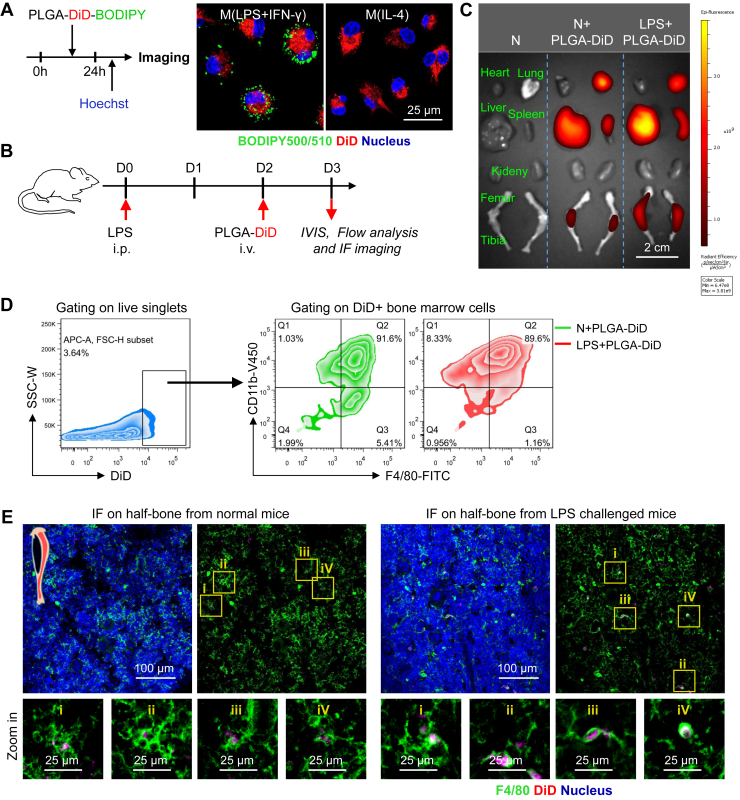
Characterization of dye-loaded PLGA NPs in vivo. A: DiD/BODIPY-loaded PLGA NPs were designed and administered into in vitro-cultured macrophages. LDs (green color, BODIPY), DiD (red color), nuclei (blue color, Hoechst 33342); the scale bar represents 25 μm. B: Experimental setup. LPS was injected into the peritoneal cavity of mice to prime inflammation. On day 2 after LPS injection, DiD-loaded PLGA NPs were intravenously injected into the tail vein of mice. On day 3 after LPS injection, organs were harvested for analysis. C: Representative IVIS images of organs from normal mice, normal mice injected with NPs, and LPS-challenged mice injected with DiD-loaded PLGA NPs. D: A flow cytometer was used to assess the macrophage purity of DiD+-gated BM cells in normal and LPS-challenged mice after DiD-loaded PLGA NP injection. E: IF of half bone was used to identify the cell type of DiD+ BM cells from normal and LPS-challenged mice after DiD-loaded PLGA NP injection; F4/80 (green color), DiD (red color), nuclei (blue color, Hoechst 33342); the scale bar represents 100 μm.

Here, we investigated the specificity of BODIPY-loaded PLGA NP delivery in vivo. Considering the absence of LDs in noninflammatory macrophages, the noninflammatory macrophages could not be labeled by BODIPY dyes and hereby could not be detected in vivo even when they uptake BODIPY-loaded PLGA NPs. For better visualization of macrophages with LDs in vivo, we prepared DiD/BODIPY-loaded PLGA NPs in which DiD dyes serve as reporter probes to label macrophages in vivo. To confirm the labeling efficacy, we first administered the DiD/BODIPY-loaded PLGA NPs into the activated BM derived-macrophages in vitro (Fig. 3A). Theoretically, the DiD dyes released from NPs will label the cells after cellular uptake, whereas the BODIPY released from NPs will label the LDs (if the cells contain LDs). Using confocal microscopy, we found that M_(LPS + IFN-γ)_ macrophages were positively stained by DiD and BODIPY, and M_(IL-4)_ macrophages were only stained by DiD (Fig. 3A). These findings reveal that these DiD/BODIPY-loaded PLGA NPs have the potential to label macrophages in vivo and distinguish the proinflammatory macrophages from others—DiD for macrophage identification and BODIPY for phenotyping.

To investigate the distribution and specificity of NPs in vivo under homeostasis and stress, intraperitoneal injection of LPS was used to prime the in vivo systemic inflammation. DiD-loaded PLGA NPs were injected on the second day after LPS injection (Fig. 3B). Using an IVIS, DiD fluorescence signals were detected in the liver, spleen, lung, and bones from normal or LPS-challenged mice on the first day after NP injection (Fig. 3C), indicating that DiD-loaded PLGA NPs were engulfed by cells in these tissues. These organs contain high numbers of macrophages (32). Next, flow cytometry and IF staining were used to clarify the identity of these DiD-positive cells. We found that most DiD-positive cells were positive for macrophage markers (CD11b and F4/80) (Fig. 3D), indicating that DiD-loaded PLGA NPs were engulfed by macrophages. Moreover, a high proportion of CD11b and F4/80-positive macrophages was stained by DiD, indicating that PLGA NPs had high in vivo labeling efficiency for macrophages (supplemental Fig. S3A). IF imaging also confirmed that DiD-stained cells were F4/80-positive macrophages in the bone, liver, and lung tissues (Figs. 3E and S3B). Overall, PLGA NPs successfully delivered the dyes to inflammatory sites and selectively stained macrophages with high specificity.

### Imaging LDs in situ helps to identify the reactivation of macrophages in biopsy tissues

**Fig. 4:**
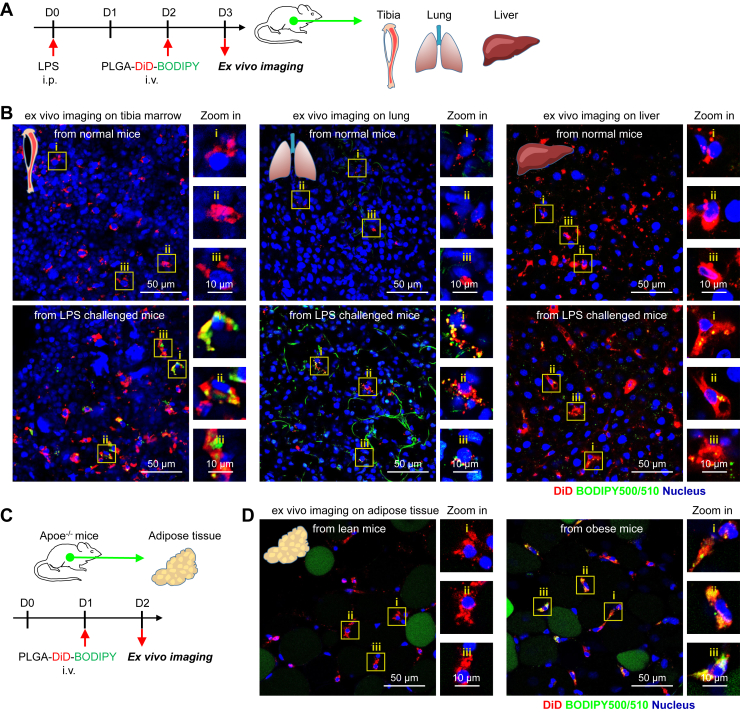
Visualization of single macrophage LD accumulation in internal organs in situ under inflammation. A: Schematic overview of experimental procedures. LPS was intraperitoneally injected to prime systemic inflammation. On day 2 after LPS injection, DiD/BODIPY-loaded PLGA NPs were intravenously injected into mice. On day 3 after LPS injection, organs were harvested for ex vivo imaging. Normal mice were used as a control. B: Ex vivo live-cell imaging on tibial marrow, lung, and liver from normal and LPS-challenged mice after NP injection. LDs (green color, BODIPY), DiD (red color), and nuclei (blue color, Hoechst 33342); the scale bar represents 50 μm. C: Schematic overview of experimental procedures. The ApoE^−/−^ mice were used to develop the animal model of obesity. DiD/BODIPY-loaded PLGA NPs were intravenously injected into mice. On day 1 after NP injection, adipose tissues were harvested for ex vivo imaging. Lean ApoE^−/−^ mice were used as a control. D: Ex vivo live-cell imaging on adipose tissues from lean and obese ApoE^−/−^ mice after NP injection. LDs (green color, BODIPY), DiD (red color), and nuclei (blue color, Hoechst 33342); the scale bar represents 50 μm.

Considering the difficulty in intravital imaging of internal organs, we isolated internal organs for ex vivo imaging after NP injection. DiD/BODIPY-loaded PLGA NPs were injected on the second day after LPS injection. One day after NP injection, tibia marrow, lung, and liver were harvested and imaged using confocal microscopy (Fig. 4A). Compared with normal mice, DiD-labeled macrophages in tibia marrow, lung, and liver of LPS-treated mice were characterized by the accumulation of BODIPY-stained LDs (Fig. 4B), indicating that macrophages undergo inflammatory activation. To identify the labeling efficiency of PLGA NPs, on the second day after LPS injection, we also harvested bones to prepare single-cell suspension and stained them with free BODIPY (supplemental Fig. S4A). Morphologically, using confocal microscopy, we found that F4/80 + CD11b + BM macrophages had LD accumulation after LPS injection relative to resting BM macrophages (supplemental Fig. S4B, C), which is consistent with the findings based on PLGA NP-mediated in vivo labeling.

To characterize the activation status of macrophages under LPS-induced stress (17), flow cytometry was performed to check for surface or intracellular markers of BM macrophages (supplemental Fig. S4A). We found that compared with that in normal mice, BM macrophages had elevated expression levels of surface markers (CD80) and functional enzymes (inducible NOS) and decreased expression levels of surface markers (CD206) after LPS stimulation (supplemental Fig. S4D, E). These data confirm that resting BM macrophages were successfully activated into the inflammatory subtype under acute systemic inflammation, which is in line with findings based on LD imaging (Figs. 4B and S4B).

Moreover, we also investigated whether this approach can label and identify the inflammatory macrophages in less severe conditions, such as obesity-induced chronic inflammation of adipose tissues. The apolipoprotein E-knockout (ApoE^−/−^) mice were used to develop the animal model of obesity by feeding high-fat diet. After DiD/BODIPY-loaded PLGA NP injection, adipose tissues were harvested and imaged using IVIS and confocal microscopy (Fig. 4C). DiD fluorescence signals were detected in adipose tissues from obese mice on the first day after NP injection (supplemental Fig. S5A), indicating that NPs were engulfed by cells in adipose tissues. IF imaging confirmed that DiD-labeled cells in adipose tissues were F4/80-positive macrophages (supplemental Fig. S5B). Compared with the macrophages in adipose tissues from lean mice, DiD-labeled macrophages in adipose tissues from obese mice exhibited high accumulation of BODIPY-stained LDs (Fig. 4D). This is in line with findings in previous reports that macrophages are repolarized into proinflammatory macrophages under high-fat diet-induced obesity (33, 34). Collectively, these results reveal that the developed approach can phenotype inflammatory macrophages in biopsy tissues under systemic or chronic inflammation

### Intravital labeling and imaging of LDs helps to identify proinflammatory macrophages in vivo

Last, to test the in vivo translational application of the developed technique, we selected BM to reconstruct the in vivo inflammatory microenvironment. BM-resident macrophages are involved in erythroid maturation and hematopoietic stem cells maintenance at homeostasis (35, 36, 37). The proinflammatory activation of BM-resident macrophages easily occur under diverse kinds of stress, resulting into various lethal BM diseases (3, 4, 38, 39, 40). Thus, in vivo analysis of the BM macrophage activation status will help enable monitoring of the therapeutic window.

**Fig. 5:**
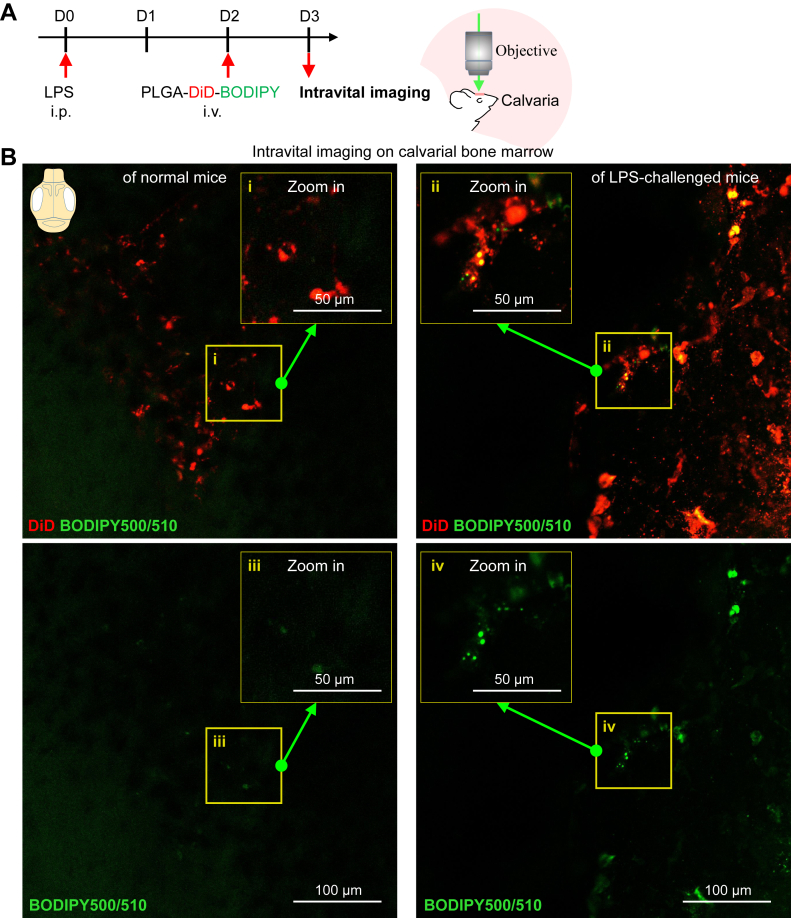
Intravital imaging of LD accumulation helps to distinguish proinflammatory macrophages from others in vivo. A: Experimental setup. LPS was intraperitoneally injected to prime inflammation. On day 2 after LPS injection, DiD/BODIPY-loaded PLGA NPs were intravenously injected into mice. On day 3 after LPS injection, mice were anesthetized for in vivo imaging. Normal mice were used as a control. B: Representative confocal images of DiD-labeled BM cells (red color) and their LDs (green color, BODIPY) in calvarial marrow of normal and LPS-challenged mice after PLGA NP injection; the scale bar represents 100 μm.

The calvarium is feasible to image without surgical manipulation and is currently the most widely used bone to study the BM microenvironment (41, 42). In this study, after LPS and DiD/BODIPY-loaded PLGA NP injection, intravital microscopy was performed in vivo on calvaria with a confocal microscope (Fig. 5A). We found that BM macrophages in normal mice were only stained by DiD, whereas BM macrophages in LPS-challenged mice were stained by DiD and BODIPY (Fig. 5B), indicating that LDs had accumulated in proinflammatory BM macrophages. Collectively, these results reveal that DiD/BODIPY-loaded PLGA NPs can be used to distinguish proinflammatory macrophages from others in their native microenvironment.

## Discussion

Various methods exist for macrophage phenotyping (43); however, many of these assays involve in vitro methodology: they cannot determine the activation status of macrophages in the native multicellular niche, limiting their preclinical and clinical translational application. The development of advanced imaging modalities has provided a feasible approach to visualize and track single cells in vivo. Dissecting the morphological features of activated macrophages is a prerequisite for distinguishing macrophage phenotypes. Increasing evidence highlights the relationships between LDs and the proinflammatory functions of macrophages (18, 19, 20). Here, we found that LD accumulation of proinflammatory macrophages provides a structural marker for future imaging studies. Along that line, we developed an optical imaging method to characterize macrophage types, using NPs that contain a combination of a lipophilic carbocyanine dye (DiD) to identify macrophages and a lipid-staining dye (BODIPY) to detect the LDs.

LD-accumulating macrophages are highly correlated with inflammatory diseases, such as atherosclerosis, obesity, diabetes, rheumatoid arthritis, and neurodegeneration (15, 44). In this study, we selected the BM, liver, lung, and adipose tissue as the tissues of interest. After NP injection, we successfully phenotype the proinflammatory macrophages from others in the biopsy tissues under inflammation. By including biopsy, the developed method could also be applied to detect macrophage phenotype in internal organs (such as BM and liver), which has translational potential for clinical diagnosis.

Intravital microscopy was performed in vivo on calvaria with a confocal microscope (41, 42). We believe that the method developed here may help noninvasively detect macrophage phenotypes in various tissues such as the mucosa and skin. Fortunately, LDs have third-harmonic generation contrasts in harmonic generation microscopy (45). By combining with multiphoton fluorescence microscopy, the identification of LD-accumulating macrophages from others without any invasive surgical manipulation will be achieved, which provides a feasible method to monitor the clinical treatment of chronic wounds. Moreover, by including endoscopy and multiphoton laser (46), the developed approach can help phenotype macrophages in atherosclerotic lesions without any exogenous labeling.

Collectively, the method developed here will help to identify the activation status and functions of macrophages in their complex and dynamic immune microenvironment. We believe that these findings will advance the functional study of macrophages and LDs in various inflammation-related diseases, such as diabetes, obesity, atherosclerosis, and chronic wounds.

## Data availability

All data are included in the article.

## Supplemental data

This article contains supplemental data.

## Conflict of interest

The authors declare that they have no conflicts of interest with the contents of this article.
