# Supplementary material for: Intravital lipid droplet labeling and imaging reveals the phenotypes and functions of individual macrophages in vivo
Source: J Lipid Res. 2022 Apr 6;63(5):100207. doi: 10.1016/j.jlr.2022.100207 (PMC9117931; doi:10.1016/j.jlr.2022.100207)
Supplement: Supplemental Figures S1–S5 [file mmc1.docx]

Supplementary Materials

Intravital lipid droplet labeling and imaging reveals the phenotypes and functions of individual macrophages *in vivo*

Yue Li^1†^, Yuwei Du^2,3,4†^, Zhengqing Xu^2,3,4^, Yuan He^5^, Ran Yao^1^, Huiran Jiang^2,3,4^, Wen Ju^2,3,4^, Jianlin Qiao^2,3,4^, Kailin Xu^2,3,4^, Tzu-Ming Liu^6*^, Lingyu Zeng^2,3,4*^

^1^School of Medical Technology, Xuzhou Medical University, Xuzhou, Jiangsu, China

^2^Blood Diseases Institute, Xuzhou Medical University, Xuzhou, Jiangsu, China

^3^Key Laboratory of Bone Marrow Stem Cell, Xuzhou, Jiangsu, China

^4^Department of Hematology, the Affiliated Hospital of Xuzhou Medical University, Xuzhou, Jiangsu, China

^5^School of Pharmacy, Xuzhou Medical University, Xuzhou, Jiangsu, China

^6^Faculty of Health Sciences, University of Macau, Taipa, Macao SAR, China

^†^These authors have contributed equally to this work and share first authorship

^*^These authors have contributed equally to this work and share last authorship

***Corresponding author:**

Lingyu Zeng, Email: [zengly2000@163.com](mailto:zengly2000@163.com); Tzu-Ming Liu, E-mail address: tmliu@um.edu.mo.

**Supplementary figures 1-5**

**
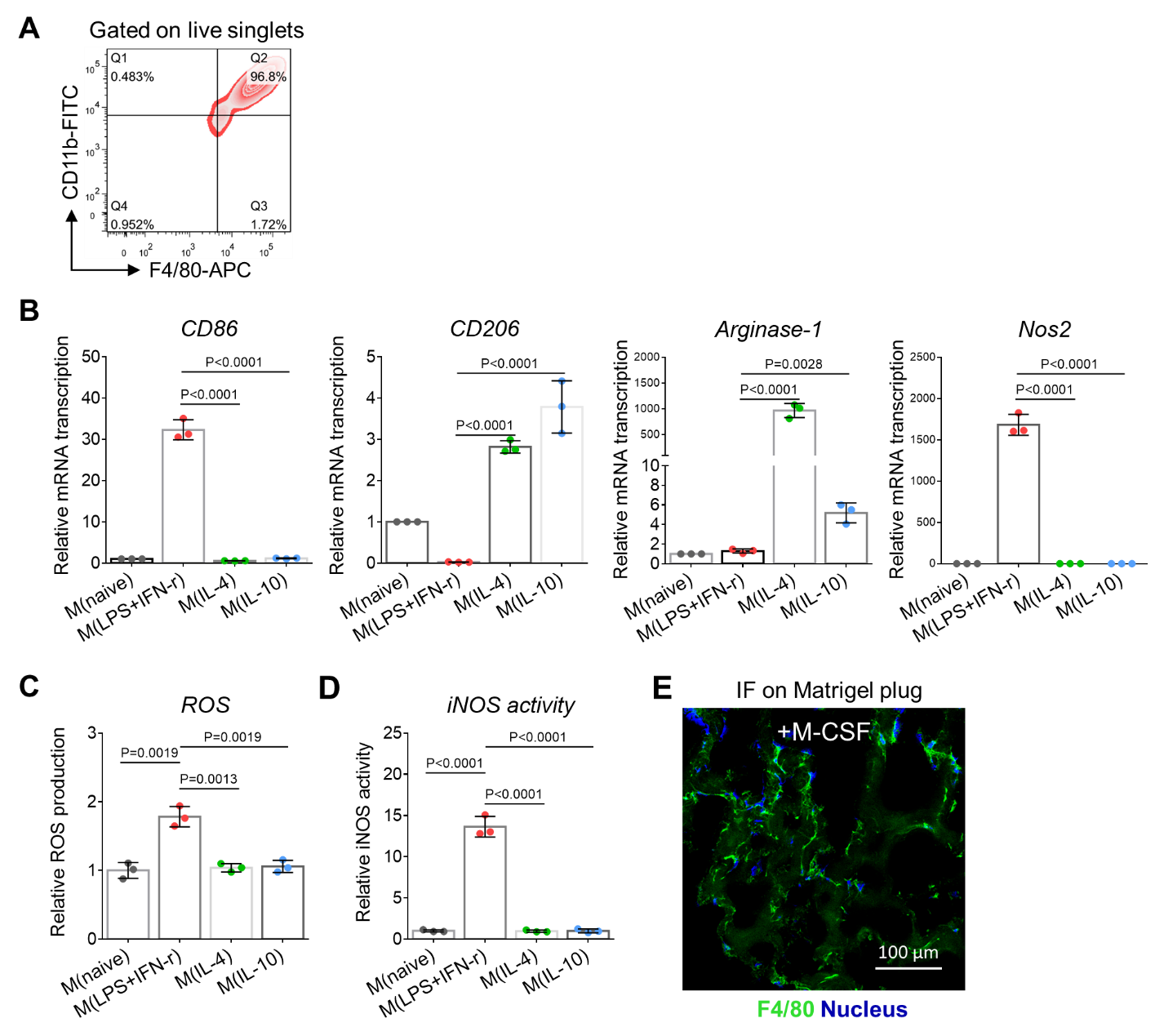
**

**Supplementary Figure 1.** Characterization of *in vitro* cultured bone marrow-derived macrophages (BMDMs) and *in vivo* Matrigel plug-recruited macrophages. (A) On the 5^th^ day after M-CSF treatment, a flow cytometer was used to assess the *in vitro* cultured macrophage purity (F4/80 and CD11b). (B) After 24 h of stimulation, RT-qPCR was used to assess the CD86, CD206, NOS2 and Arginase-1 gene expression of activated macrophages. (C-D) The production of reactive oxygen species (ROS) and the activity of inducible nitric oxide (NO) synthase of activated macrophages were measured using DCFH-DA and DAF-FM DA fluorescent probes, respectively. (E) IF imaging of F4/80 expression (green) of fixed cells in implanted Matrigel plugs. The nuclei are stained with Hoechst 33342 (blue). Scale bar, 100 µm.

**
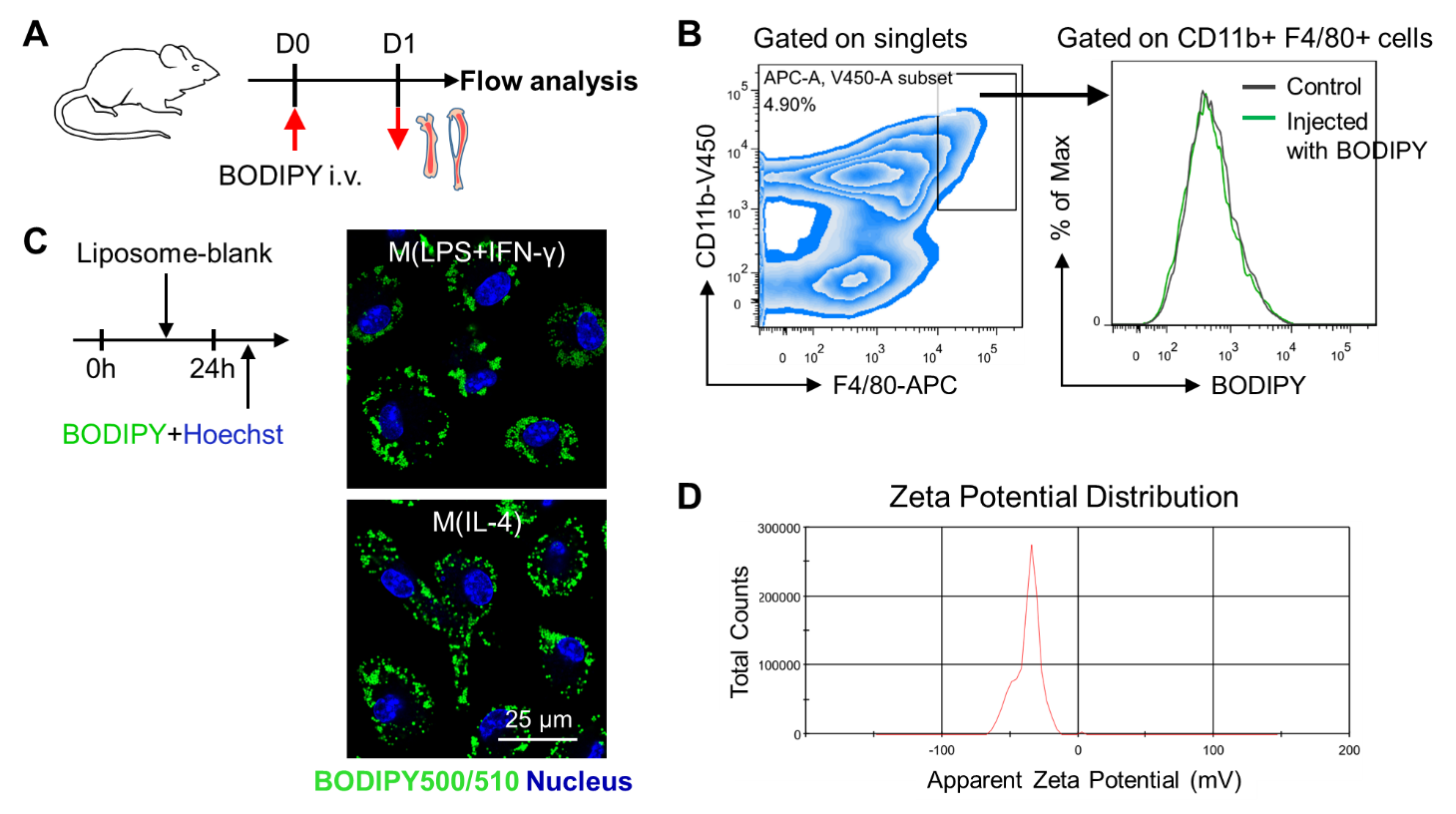
**

**Supplementary Figure 2.** Characterization of BODIPY-loaded NPs *in vitro*. (A) Experimental protocol. To label the LDs of bone marrow-resident macrophages, free BODIPY resuspended in PBS was intravenously injected into mice; then, bones were harvested to prepare a single-cell suspension for flow cytometry. (B) On the first day after BODIPY injection, a flow cytometer was used to assess the BODIPY fluorescence intensity of F4/80+ CD11b+ gated bone marrow cells in normal mice and BODIPY-injected mice. (C) To check the effect of liposome engulf on intracellular LD formation, blank liposomes were administrated into *in vitro* cultured macrophages, then the cells were counterstained by free BODIPY and Hoechst 33342. LDs (green color, BODIPY) and nuclei (blue color, Hoechst 33342); Scale bar, 25 µm. (D) The zeta potential distribution of BODIPY-loaded PLGA NPs diluted in water.

**
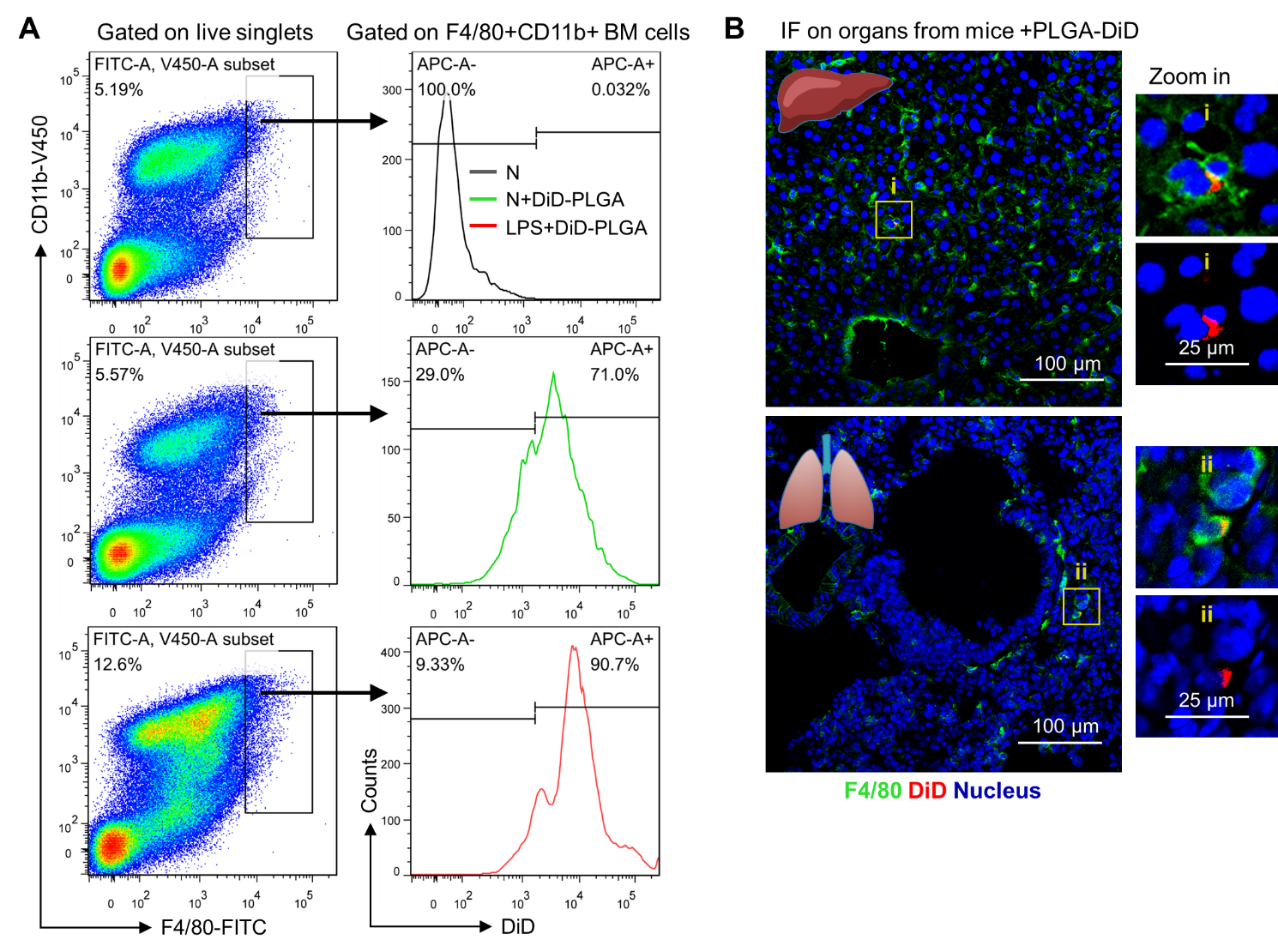
**

**Supplementary Figure 3.** Labeling specificity of DiD-loaded PLGA NPs in mice. (A) After DiD-loaded PLGA NP injection, a flow cytometer was used to assess the DiD fluorescent intensity of F4/80+CD11b+ gated bone marrow cells in normal mice (N), normal mice injected with NPs (N+PLGA-DiD), and LPS-challenged mice injected with NPs (LPS+PLGA-DiD). (B) IF of organs (liver and lung) was used to identify the cell type of DiD+ cells from mice after DiD-loaded PLGA NP injection; F4/80 (green color), DiD (red color), nuclei (blue color, Hoechst 33342); Scale bar, 100 µm.


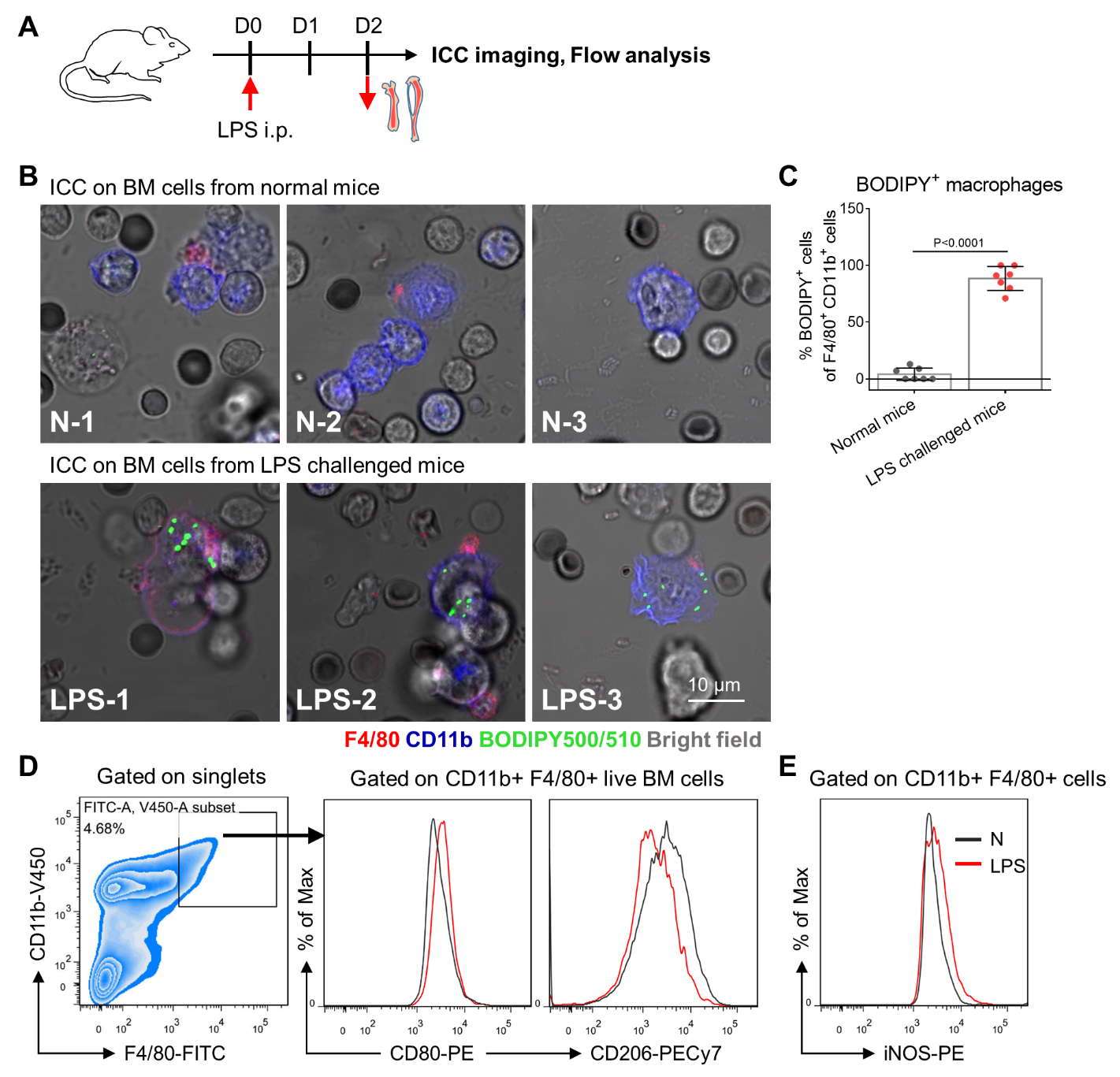


**Supplementary Figure 4.** Analysis of the re-activation of BM macrophages under inflammatory stimulation. (A) Experimental set-up. LPS was injected into the peritoneal cavity of mice to prime systemic inflammation. On day 2 after LPS injection, bones were harvested to prepare single BM cell suspension for flow cytometry and immunocytochemistry (ICC) imaging. Normal mice were used as a control. (B) Representative fluorescence images of LDs in BM macrophages after ICC and free BODIPY staining. Cell morphology (gray color, bright field), LDs (green color, BODIPY), F4/80 (red color), CD11b (blue color). Scale bar, 10 µm. (C) Quantification of BODIPY+ (LD-containing) macrophages in F4/80 and CD11b-positive cells. (D-E) A flow cytometry was used to assess surface markers (CD80 and CD206) and intracellular marker (iNOS) expression of the F4/80+ CD11b+ BM macrophages from normal and LPS-challenged mice. All data shown were representative of two or three independent runs of experiments.

**
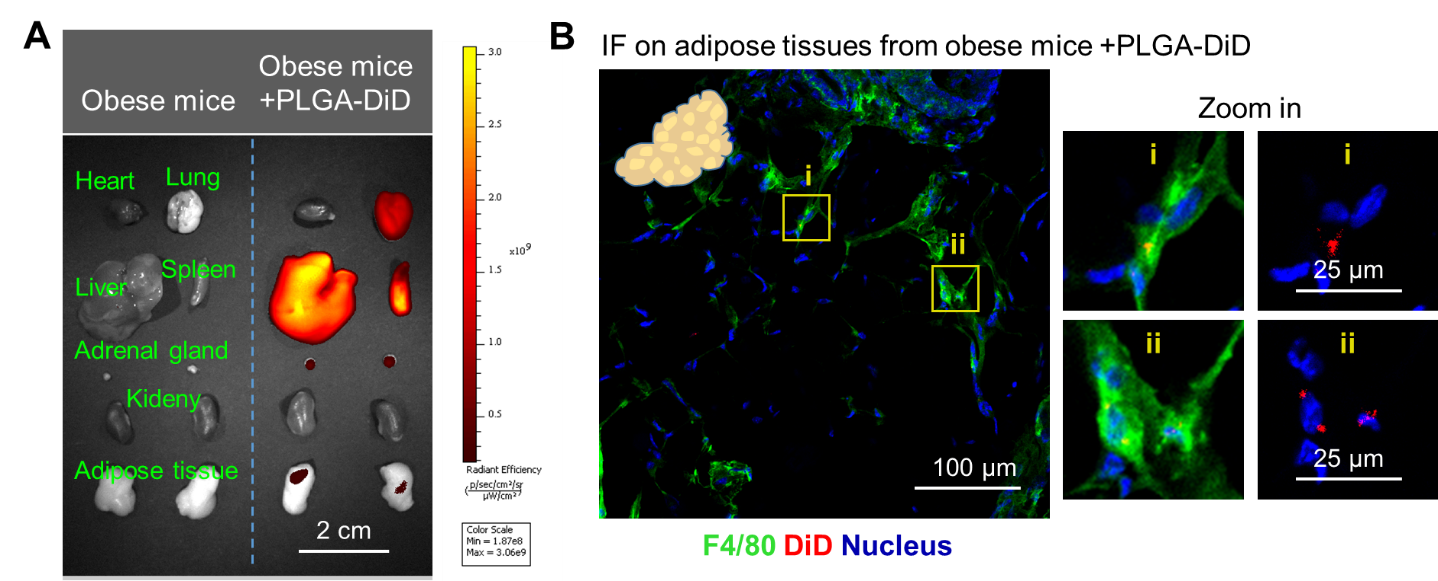
**

**Supplementary Figure 5.** Characterization of DiD-loaded PLGA NPs in mice with diet-induced obesity. (A) In vivo imaging system (IVIS) was used to analyze the *in vivo* distribution of DiD-loaded PLGA NPs after injection. Representative IVIS images of organs (one day post-NP injection) from obese ApoE^-/-^ mice, and obese ApoE^-/-^ mice injected with NPs. (B) IF of organs (adipose tissues) was used to identify the cell type of DiD+ cells from mice after DiD-loaded PLGA NP injection; F4/80 (green color), DiD (red color), nuclei (blue color, Hoechst 33342); Scale bar, 100 µm.
